# Lutein levels in arterial cord blood correlate with neurotrophic calcium binding S100B protein in healthy preterm and term newborns

**DOI:** 10.1186/s13052-022-01276-9

**Published:** 2022-05-28

**Authors:** Simonetta Picone, Alberto Ritieni, Giulia Graziani, Piermichele Paolillo, Ebe D’Adamo, Valentina Botondi, Daniele Panichi, Sara Torresi, Daniela David, Armando di Ludovico, Francesco Chiarelli, Diego Gazzolo

**Affiliations:** 1 Neonatology and Neonatal Intensive Care Unit, Policlinico Casilino General Hospital, Rome, Italy; 2 Department of Pharmacy, Federico II Naples University, Naples, Italy; 3 Neonatal Intensive Care Unit, G. d’Annunzio University, Chieti, Italy; 4 Department of Pediatrics, University of Chieti, Chieti, Italy

**Keywords:** Newborns, Cord blood, Lutein, S100B, Biomarkers, Brain development

## Abstract

**Background:**

S100B is an established biomarker of brain development and damage. Lutein (LT) is a naturally occurring xanthophyll carotenoid mainly concentrated in the central nervous system (CNS), but its neurotrophic role is still debated. We investigated whether LT cord blood concentrations correlate with S100B in a cohort of preterm and term healthy newborns.

**Methods:**

We conducted a prospective study on the distribution of LT and S100B in arterial cord blood of healthy preterm (*n* = 50) and term (*n* = 50) newborns.

**Results:**

S100B and LT showed a pattern of concentration characterized by higher levels (*P* < 0.01, for all) at 33-36 weeks gestation (GA) followed by a progressive decrease (*P* < 0.01, for all) from 37 onwards with a dip at term. Both S100B and LT were gender-dependent with significantly (*P* < 0.01, for all) higher levels in females in preterm and term groups. S100B (*R* = 0.68; *P* < 0.001) and LT (*R* = 0.40; *P* = 0.005) correlated with GA at sampling. A positive significant correlation (*R* = 0.87; *P* < 0.001) between S100B and LT was found.

**Conclusions:**

The present data showing a correlation between S100B and LT supports the notion of a LT trophic role in the CNS. Further investigations in high-risk infants are needed to elucidate LT involvement in the pathophysiological cascade of events leading to CNS development and damage.

## Background

S100B is a brain-specific acidic calcium binding protein of the EF-hand family mainly concentrated in glial cells and in neuronal sub-populations [1]. There is evidence that the protein is a consolidated marker of central nervous system (CNS) growth and damage mimicking a Janus face: when secreted, S100B is believed to have paracrine/autocrine trophic effects at physiological (nanomolar) concentrations, and neurotoxic effects at higher (micromolar) concentrations [2].

In different biological fluids (i.e. amniotic, cerebrospinal, blood, urine, saliva and milk) of fetuses, newborns and pediatric patients S100B correlates with clinical, laboratory and radiologic parameters suggestive of CNS development and damage [2–9].

In the last decade, Food and Drugs Administration (FDA), European Medical Agency (EMA) and more recently the National Institute of Health (NIH) supported investigations on neurobiomarkers (NB) in the perinatal period. The aim was to encourage the integration of NB in drug development and their appropriate use in clinical practice, promoting NB qualification programs [10].

Among different new NB, to date still matter of investigation Lutein (LT) is a naturally xanthophyll carotenoid not synthesized by humans. In biological fluids and tissues LT concentration depends on daily dietary intake since it is found at high levels in fruits and vegetables (i.e. spinach and kale) [11–14]. In the perinatal period, LT has been shown to be highly concentrated in different biological fluids (blood and human milk) and in CNS specific areas (i.e. neuro-retinal, frontal, occipital cortex and hippocampus) [15–18]. In this regard, LT has been shown to be involved in CNS development through a not still understood mechanism [19, 20]. LT trophic role has been suggested by its clinical and biochemical correlation: the former with gestational age (GA) and gender in healthy preterm/term newborns; the latter with a consolidated brain growth factor such as Activin A [20].

Therefore, in the present study we aimed at investigating whether LT arterial cord blood levels correlated with S100B in healthy preterm and term infants, thus supporting its CNS trophic role.

## Materials and methods

### Population

The local Ethics Committees approved the study protocol and informed and signed consent was obtained from all parents of patients.

**Fig. 1:**
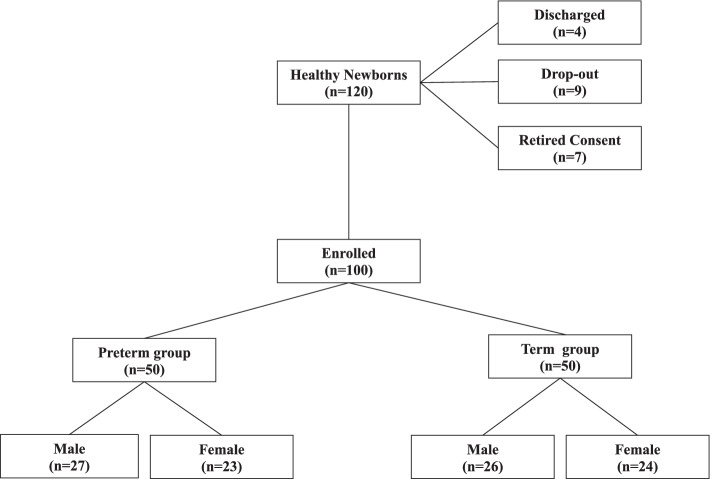
Flow chart showing patient recruitment

We recruited 100 women with consecutive healthy pregnancies (preterm *n* = 50; term *n* = 50), delivering between 33^+6d^ and 41^+6d^ weeks of GA (Fig. 1).

GA was determined by clinical data and by ultrasound scan performed in the first trimester. Appropriate growth was defined as follows: i) a biparietal diameter and abdominal circumference between 10^th^-90^th^ centiles in agreement with Campbell and Thoms [21], and ii) birth weight (BW) between 10^th^-90^th^ centiles according to our population standards, after correction for the mother’s height, weight, parity, and the sex of the newborn [22]. Preterm and term newborns were classified when they were born before or after 37 GA, respectively. We include into the study only preterm and term infants showing the following perinatal outcomes: no maternal illness; no signs of fetal distress; pH > 7.2 in cord or venous blood; and Apgar scores at 1-5 min > 7.

Exclusion criteria were: the presence of any maternal CNS illness, multiple pregnancies, intrauterine growth retardation, gestational hypertension, diabetes and infections, any fetal malformations, chromosomal abnormalities, perinatal asphyxia and dystocia.

Arterial cord blood samples were collected at birth to assess standard laboratory monitoring parameters, S100B and LT. All samples collected for NB measurement were centrifuged at 900 g (S100B) and 2500 g (LT), respectively, and stored at − 70 °C.

### Neurological examination

Neurological examination was performed daily during hospital staying according to a qualitative approach by Prechtl [23] that assigned each infant to one of three diagnostic groups: normal, suspect or abnormal. An infant was considered abnormal when one or more of neurological syndromes such as hyper- or hypokinesia, hyper- or hypotonia, hemisyndrome, apathy and hyperexcitability syndromes were present. An infant was classified as suspect in absence of a defined syndrome or when only isolated signs were present. Neurological examination was performed by a single examiner who was blind to NB results.

### S100B measurement

S100B levels were measured using an immunoluminometric assay (Liaison S100, Dietzenbach, Germany) according to the manufacturer’s instructions. The detection limit of the assay was 0.02 μg/L, the coefficient of variation was ≤2.8% within-assay and ≤ 5.3% interassay for concentrations ranging between 0.09 and 18.9 μg/L.

### Lutein measurement

LT extraction and high-performance liquid cromatography (HPLC) analysis were performed using analytical conditions as previously reported [18, 20]. Briefly, an aliquot of 500 μL of serum was treated with 500 μL of ethanol (1% BHT) to precipitate proteic pellet. Hydroalcoholic fraction and pellet were separately extracted twice with 500 μL of hexane. Hexanic fractions, containing serum carotenoids, were combined, evaporated under nitrogen and solubilized in 50 μL of chloroform before HPLC analysis. Chromatographic separation of LT was performed on a LC-10 AD Shimadzu HPLC system equipped with binary pump and a column compartment, coupled to a UV-diode array detector.

Separation was performed on a Develosil 5 μm RP-AQUEUOUS C30, 250 × 4,6 mm column (Phenomenex Torrance, CA, USA) using chromatographic conditions previously reported [18]. Quantification of LT was done by the external standard method using a calibration curve built with LT as reference standard. The limit of detection was 0.017 nmol/mL.

The identification of LT and its metabolite was performed by liquid chromatography coupled to a tandem mass spectrometer. The chromatographic separation was performed by HPLC connected with two micro-pumps 200 (Perkin Elmer, Carlsbad, CA, USA), using the same column and the same chromatographic conditions described for HPLC analysis.

The API 3000 tandem mass spectrometer (API 3000, Applied Biosystem, Waltham, MA, USA) equipped with an atmospheric pressure chemical ionization source (APCI) was used for mass spectrometry analysis. The optimum settings of the mass spectrometer were: probe temperature 500 °C, the nebulizer current 4 μA, declustering potential 45 V and focusing potential 300 V.

### Cerebral ultrasound

CUS was performed during the first 72 h of life or at discharge from hospital in all the study population. Recordings were performed by real-time ultrasound machine (Acuson 128SP5 Mountain View CA, USA) at the predetermined monitoring time-points. A single examiner who did not know the results of the cord blood test and clinical data reviewed images. Cerebral haemorrhage was classified according to Papile et al. criteria [24].

### Monitoring parameters

In all recruited infants pulsed arterial oxygen saturation (SaO_2_) and the main laboratory parameters such as red blood cell count (RBC); hemoglobin blood concentrations (Hb); hematocrit rate (Ht); venous blood pH; partial venous carbon dioxide pressure (pCO_2_); partial venous oxygen pressure (pO_2_); base excess (BE); blood ions were recorded at the admission into the study.

## Statistical analysis

For sample size calculation, we used changes in S100B arterial cord blood concentrations in healthy preterm and term infants at birth as the main parameter [25]. We assumed a decrease of 0.5 standard deviation (SD) in S100B to be clinically significant. Considering an α = 0.05 and using a two-sided test, we estimated a power of 0.95, recruiting 45 preterm and 45 term infants. We added *n* = 5 infants per group to allow for dropouts, cross-over and consent retirement (Fig. 1).

Clinical data is reported as mean and SD. Biochemical data is reported as median and interquartile centiles. The results of fetal and neonatal monitoring parameters were compared between groups by the two-sided Mann–Whitney U test and by Kruskal–Wallis one-way ANOVA followed by the Dunn post-hoc test when the data did not follow a Gaussian distribution. Comparisons between proportions were performed with the Fisher exact test. Linear regression analysis was performed for correlations between S100B, LT and GA, respectively. Statistical analysis was performed using Sigma Stat 3.5 (GmBH, Germany). A value of *P* < 0.05 was considered significant.

## Results

Table 1 shows maternal and perinatal characteristics in the term and preterm newborns at admission into the study. In particular, maternal age, delivery mode and gender were comparable between the two groups (*P* > 0.05, for all).

**Table 1:** Perinatal characteristics in preterm and term newborns

Parameters	Preterm (***n*** = 50)	Term (***n*** = 50)
Maternal Age *(y)*	31 (3)	32 (2)
*Mode of delivery, n (%)*		
Caesarean	10 (20)	5 (10)
Vaginal	40 (80)	45 (90)
Gender (male/female)	27/23	26/24
BW (g)	2615 (377)	3390 (312)^*^
GA (wks)	35 (1)	40 (2)^*^
*Apgar score > 7 n (%)*		
At 1 min	50 (100)	50 (100)
At 5 min	50 (100)	50 (100)
SaO_2_ (%)	95 ± 3	96 ± 2
*Laboratory parameters*		
RBC (10^6^/mm^3^)	3.87 ± 0.2	3.95 ± 0.03
Hb (g/dL)	13.3 ± 0.02	13.5 ± 0.01
Ht (%)	40.1 ± 0.04	40.5 ± 0.02
Venous blood pH	7.32 ± 0.01	7.35 ± 0.06
pCO_2_ (mmHg)	42.6 ± 1.5	41.3 ± 1.5
pO_2_ (mmHg)	43.1 ± 1.8	45.2 ± 0.7
Base excess	− 0.4 ± 0.1	−0.3 ± 0.9
Na^+^ (mmol/L)	137 ± 0.3	139 ± 0.4
K^+^ (mmol/L)	4.0 ± 0.1	4.2 ± 0.1
Ca^++^ (mmol/L)	1.15 ± 0.02	1.13 ± 0.01
*Prechtl score (normal/total)*		
Normal	50 (50)	50 (50)
Suspect	0 (50)	0 (50)
Abnormal	0 (50)	0 (50)
*Cerebral ultrasound (normal/total)*	50 (50)	50 (50)

Data are given as mean ± (SD)

*Abbreviations*: *GA* gestational age, *BW* Birth-weight, *SaO*_*2*_ arterial oxygen saturation, *RBC* Red blood cell count, *Hb* Hemoglobin, *Ht* Hematocrit rate;*\ pCO*_*2*_ partial venous carbon dioxide pressure, *pO*_*2*_ partial venous oxygen pressure

^*^P < 0.05

As expected, significant differences (*P* < 0.001, for both) were observed between preterm and term newborns regarding GA and BW. No significant differences (*P* > 0.05, for all) were detectable between studied groups regarding Apgar scores at 1st and 5th minutes, SaO_2_ and the main laboratory parameters (RBC, Hb, Ht, venous blood pH; pCO_2_, pO_2_, BE and blood ions).

Neurological examination and cerebral ultrasound patterns did not differ between groups. Moreover, at discharge from hospital, no overt neurological syndrome was detectable in all infants admitted to the study.

### S100B protein measurements

S100B arterial blood levels were detectable in all the samples collected. S100B pattern of concentration was characterized by higher levels in the early GA (*P* < 0.01, for all) with a peak at 33-36 GA (S100B median: 1.51 μg/mL; 25^th^–75^th^ centile: 1.12-1.90 μg/mL) and by a progressive decrease, with a dip at 41 GA (S100B median: 0.60 μg/mL; 25^th^–75^th^ centile: 0.33-0.75 μg/mL). Moreover, S100B has been found to be GA dependent (*R* = − 0.68; *P* < 0.001).

**Fig. 2:**
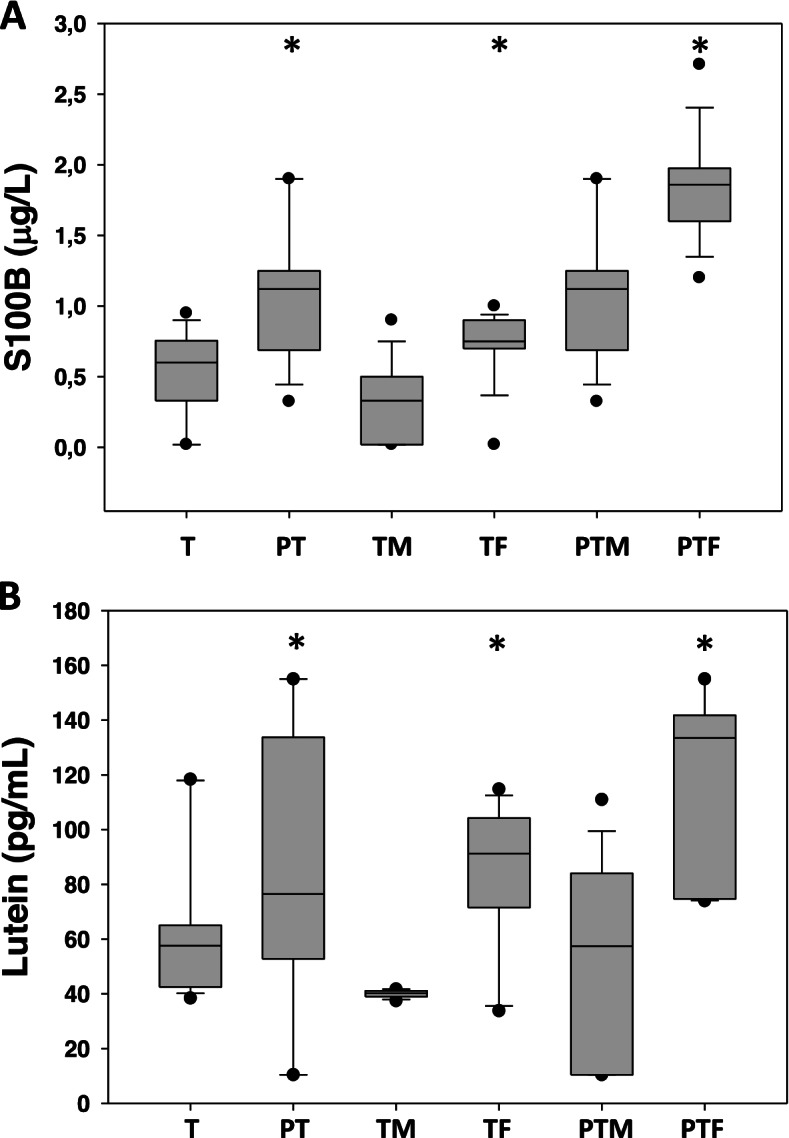
Panel **A** S100B (μg/L) arterial cord blood concentrations in term (T) and preterm (PT) newborns and when corrected for gender (male: M; female: F). Data are given as median and 5^th^–95^th^ centiles. (*) *P* < 0.01. Panel **B** Lutein (pmol/mL) arterial cord blood concentrations in term (T) and preterm (PT) newborns and when corrected for gender. Data are given as medians and 5^th^–95^th^ centiles. (*) *P* < 0.01

When S100B levels were corrected for gender, we found higher (*P* < 0.01, for all) protein levels in females than males both in preterm and term infants (Fig. 2, panel A).

### Lutein measurements

LT arterial blood levels were detectable in all the samples collected. LT showed a pattern of concentration characterized by higher levels in the early GA (*P* < 0.01, for all) with a peak at 33-36 GA (LT median: 82.06 pmol/mL; 25^th^–75^th^ centile: 57.45–133.50 pmol/mL), followed by a progressive decrease from 37 GA onwards, with the lower dip at 42 GA (LT median: 57.60 pmol/mL; 25^th^–75^th^ centile: 42.50–65.07 pmol/mL). Moreover, LT has been found to be GA dependent (*R* = − 0.40; *P* = 0.005). When LT levels were corrected for gender, we found higher (*P* < 0.01, for all) LT levels in females than males both in preterm and term infants (Fig. 2, panel B).

### S100B and lutein correlation

In Table 2 S100B and LT correlations are reported. There were significant positive correlations (*P* < 0.05, for all) between S100B and LT when considered total populations and when sub-groups for GA and gender.

**Table 2:** Correlation between S100B protein and Lutein arterial cord blood levels (LT) at different gestational ages and after correction for gender

Parameter	R	***P***
S100B total vs LT total	0.65	< 0.001
S100B term vs LT term	0.87	< 0.001
S100B term male vs LT term male	0.84	< 0.001
S100B term female vs LT term female	0.65	< 0.001
S100B preterm vs LT preterm	0.77	< 0.001
S100B preterm male vs LT preterm male	0.80	< 0.001
S100B preterm female vs LT preterm female	0.42	0.04

## Discussion

In the last decade there has been an emerging request of new diagnostic tools to include in daily clinical practice for early detection of cases at risk for perinatal brain injury [2–4]. On this light, FDA, EMA and more recently NIH approved the inclusion of NB such as S100B, Ubiquitin carboxyl-terminal hydrolase L1a and glial fibrillary acidic protein (G-FAP) in clinical protocols of adult and pediatric diseases such as traumatic brain injury [26].

In the perinatal period, there is growing evidence that the pathophysiological cascade of events involved in CNS development and damage are still not fully elucidated and are subjects of investigation. More recently, it has been shown that among a series of NB, S100B appears the only one fulfilling the majority of the criteria requested by FDA, EMA and NIH statements [10, 19, 27].

In the present study, we provide evidence that, in preterm and term healthy infants, arterial cord blood LT levels correlated with a consolidated NB of CNS development/damage namely S100B protein. Furthermore LT, as S100B, were GA- and gender-dependent with higher LT and S100B levels detectable in the early weeks of the third trimester of gestation (i.e. 33-36 wks) and lower in the near term.

The fact that both LT and S100B were GA and gender-dependent agrees with previous observations [5–9, 18, 20]. S100B trophic role is not surprising since the protein has been recently shown to fulfill all FDA, EMA, and NIH criteria. Nonetheless, LT trophic role is far to be fully elucidated. Results showed that LT was higher in the late preterm period (i.e. 33^+6d^-36^+6d^ wks) mimicking S100B pattern. The finding deserves further consideration bearing in mind that at this time period the brain volume, weight and structure are at their highest growing level [28–31]. In particular: i) magnetic resonance imaging (MRI) patterns were suggestive of the timing and duration of different myelinisation processes at the stage under investigation [30], ii) the assessment in biological fluids of NB such as S100B, activin A, G-FAP were in agreement with MRI patterns and with the biochemical, morphological and electrophysiological maturation of the CNS [2–4, 19, 28, 31], and iii) near infrared spectroscopy patterns in the late preterm period, in healthy infants, showed an improved oxygenation status and increased tissue function suggestive of CNS development [32–34]. On the basis of the present findings, it is reasonable to support a LT CNS trophic role. The explanation may reside in LT site of concentration, metabolic and signal transmission actions. In particular LT: i) is mainly located in CNS areas crucial for learning and memory [16, 17], ii) correlates with, metabolites (i.e. 1-octadecanol, phosphate, NADH) involved in energy pathways leading to the peak of myelinisation during CNS development [17, 35, 36], iii) correlates with fatty acids and lysophospholipids involved in cortical development and folding, oligodendrocyte maturation and intracellular and cell-cell signalling [36–38], and iv) correlates with neurotransmitters (i.e. γ-aminobutyric acid, aspartate) involved in the main CNS developmental cascade of events (i.e. modulation of neuronal proliferation and maturation, neurite outgrowth, synapse formation, neurotransmission) [17, 39–43]. On the other hand, S100B: i) is mainly concentrated in the CNS where is located in glial and neuronal cells [2–4], ii) interacts with dopamine D2 receptor that belongs to the G protein-coupled receptor family with seven transmembrane domains and is widely distributed in neurons of the CNS [44], iii) extracellularly, it binds to receptor for advanced glycation end products, which activates several intracellular signalling pathways such as regulations of phosphorylation mediated by protein kinases, modulation of enzymatic activity, maintenance of cell shape and motility, influence of some signal transduction pathways, and promotion of calcium homeostasis [45, 46], and iv) depending on the concentration secreted, it exerts either trophic or toxic effects. At lower concentrations (nanomolar), S100B is thought to stimulate neuronal growth and enhancement of neuronal survival during development, while at higher concentrations (micromolar), S100B may have deleterious effects by increasing expression of the pro-inflammatory cytokine IL-6 and inducing apoptotic neuronal death [2–4]. On the basis of the present findings, it is reasonable to argue that LT and S100B are involved in a cascade of events modulating CNS development and neuroprotection.

Lastly, in the present series we also showed that LT and S100B are gender dependent. The finding deserves further consideration bearing in mind the different fetal/neonatal: i) pattern of CNS development in the two sexes, ii) growth, metabolic, hormonal developmental steps of maturation and, iii) biochemical and NB patterns of concentration at the stage under investigation [2–4, 6–8].

Finally, we recognize that the present study has several limitations, such as: i) the small population recruited, ii) the need of an empowerment in LT measurement technique in terms of reproducibility, and iii) longitudinal NB monitoring. Further investigations aimed at addressing the aforementioned issues are eagerly awaited.

## Conclusions

In conclusion, the present results showing a correlation between LT and S100B support the notion of a potential LT neurotrophic role and open the way to further investigations aimed at promoting LT in a panel of trustable biomarkers of CNS development/damage.

## Data Availability

The deidentified individual participant data will not be made available.

## Abbreviations

CNS: Central nervous System
FDA: Food and Drug Administration
EMA: European Medicine Agency
NIH: National Institute of Health
NB: Neurobiomarkers
LT: Lutein
GA: Gestational age
BW: Birth Weight
HPLC: High Performance liquid Cromatography
APCI: Atmospheric Pressure Chemical Ionization
CUS: Cerebral Ultrasound
SaO_2_: Pulsed Arterial Oxygen Saturation
RBC: Red Blood Cell Count
Hb: Hemoglobin Blood Concentration
Ht: Hematocrit Rate
pCO_2_: Partial Venous Carbon Dioxide Pressure
pO_2_: Partial Venous Oxygen Pressure
BE: Base Excess
SD: Standard Deviation
G-FAP: Glial fibrillary acidic protein
MRI: Magnetic Resonance Imaging
